# Exertion-Induced Intrathecal Haematoma in Undiagnosed Von Willebrand Disease: A Case of Acute Spinal Cord Compression

**DOI:** 10.7759/cureus.88437

**Published:** 2025-07-21

**Authors:** Zain Habib, Muhammad Umer Rasool, Azeem Ahmed, Rama Mohan

**Affiliations:** 1 Department of Trauma and Orthopaedics, Manchester University NHS Foundation Trust, Manchester, GBR; 2 Department of Orthopaedics, North Manchester General Hospital, Manchester, GBR; 3 Department of Trauma and Orthopaedics, North Manchester General Hospital, Manchester, GBR

**Keywords:** decompressive laminectomy, orthopaedic emergency, spinal decompression, spontaneous spinal subdural hematoma, von willebrand diseases

## Abstract

Intrathecal haematoma is a rare and potentially devastating cause of acute spinal cord compression. In the absence of trauma, anticoagulation, or a known bleeding disorder, diagnosis can be significantly delayed. This report presents an unusual case of an intrathecal haematoma precipitated by exertion in a previously undiagnosed case of von Willebrand disease. A 28-year-old previously healthy female presented with a sudden onset of thoracolumbar back pain following a chest press exercise. Initial assessment revealed no neurological deficits; however, she declined MRI and blood tests due to severe needle phobia and self-discharged. She re-presented hours later with progressive paraplegia, urinary incontinence, and sensory loss. MRI showed an intrathecal haematoma, prompting emergency surgical decompression. Intra-operative findings confirmed a dense intrathecal haematoma compressing the spinal cord. Coagulation studies postoperatively revealed von Willebrand disease, with prolonged activated partial thromboplastin time and low VWF antigen (31.3), VWF:RiCoF (10), and factor VIII (47.6). The patient achieved near-complete neurological recovery by post-operative day 34 and received factor replacement therapy and enhanced haematological follow-up. This case illustrates how exertion hypothetically may provoke spinal bleeding in individuals with latent coagulopathy, potentially via increased intrathoracic pressure. It also highlights the diagnostic challenges of intrathecal haematomas, particularly in the absence of trauma or known risk factors. Notably, the initial MRI report misclassified the haematoma location, corrected only via interdisciplinary imaging review intra-operatively. Intrathecal haematomas should be considered in the differential diagnosis for acute spinal cord compression, particularly when red flag symptoms are present. A detailed history, high index of suspicion, and dynamic radiological reassessment are critical. Early surgical intervention can lead to excellent neurological outcomes, even in severe cases.

## Introduction

Spinal haematomas are rare entities, with intrathecal haematomas being particularly uncommon. In the absence of trauma, anticoagulation, or known coagulopathy, such presentations pose a diagnostic dilemma with differentials including transverse myelitis, disc herniation, and cauda equina [1,2]. Atypical presentations in spinal haematomas often impede early diagnosis, with delayed intervention being a risk factor for poor prognosis. We present a rare case of an intrathecal haematoma caused by exertional strain in a patient with a previously undocumented diagnosis of von Willebrand disease, a coagulopathy associated with bleeding risk. Patients with spontaneous spinal haematomas are often found to have an underlying coagulopathy in up to 48% of cases; however, reports specifically linking von Willebrand disease to spontaneous intrathecal haematomas are exceedingly scarce in the literature. To our knowledge, only a handful of such cases have been described, and exertional strain as a precipitating factor remains even more unusual [3,4].

## Case presentation

A 28-year-old right-hand dominant female school teacher, fit and healthy with no past medical history and no known coagulopathies, presented to the emergency department (ED) in gym clothes after experiencing sudden thoracic and lumbar back pain radiating bilaterally, predominantly on the right, following a chest press exercise.

On assessment, she exhibited no neurological deficits. Digital rectal examination was declined. She was independently mobile and reported no bladder or bowel symptoms. She exhibited a normal lower limb neurological examination, with normal tone, power, reflexes and coordination. A post-void bladder scan showed a volume of 416 mL. This was immediately recognised as a red flag and more than 100 mL above the upper limit of normal. The significance of this was explained to the patient, and an MRI of the spine and blood tests were advised. However, the patient refused and self-discharged, due to being highly needle-phobic as well as feeling that a potential admission for an emergency MRI scan was superfluous. She was counselled on further red-flag symptoms, signed self-discharge paperwork, and her departure was documented and escalated.

She re-presented hours later with altered sensation below the bust line to the toes bilaterally, exhibiting a bilateral lower limb weakness, with a Medical Research Council (MRC) power grading of 3/5 in myotomes L2-S1, perianal numbness and urinary incontinence. An urgent MRI was completed, showing a spinal haematoma, initially reported as an epidural haematoma at T3-T4, with signal changes at T9-T11. She was blue-lighted to a spinal centre, where repeat imaging and clinical deterioration prompted emergency surgery. Prior to emergency surgery, the patient had become progressively paraplegic as well as doubly incontinent.

**Figure 1:**
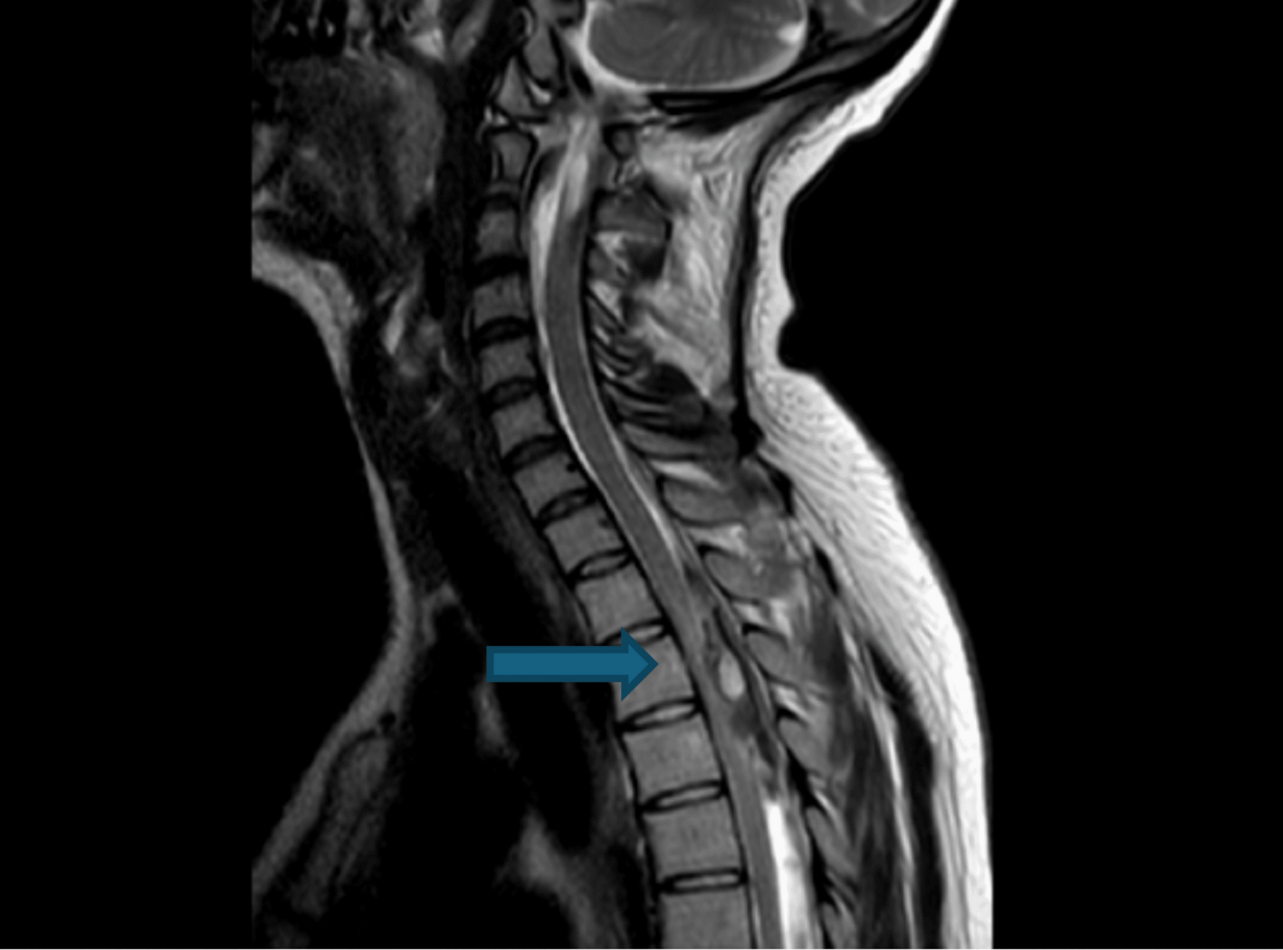
Sagittal T2-weighted MRI showing intrathecal haematoma most prominent at T3-T4. The blue arrow indicates the location of the intrathecal spinal haematoma on sagittal MRI.

**Figure 2:**
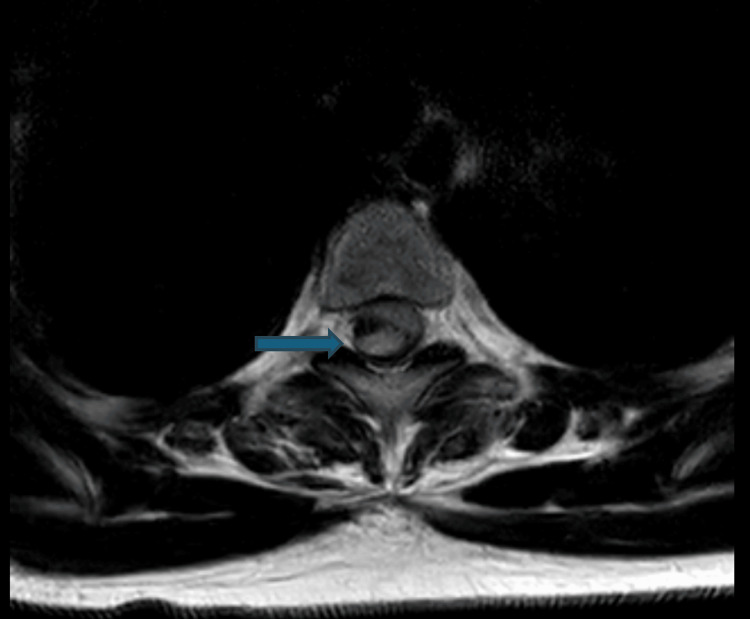
Axial T2-weighted MRI at level T3-T4 showing intrathecal haematoma. The blue arrow indicates the location of the intrathecal spinal haematoma on axial MRI.

Intra-operatively, no extradural haematoma was found. Intra-operative ultrasound indicated a dense haematoma within the thecal sac. A real-time review by an on-call neuroradiologist revealed the haematoma to be intrathecal. MRI images depicting this are shown in Figures 1, 2. Figure 1 shows a sagittal T2-weighted MRI image depicting a hyperintense mass compressing the spinal cord at T3-T4, consistent with subacute intrathecal blood. Figure 2 shows an axial T2-weighted MRI image depicting a hyperintense mass within the spinal cord, again consistent with subacute intrathecal haematoma. Interestingly, it resembles the described "inverted Mercedes-Benz sign" with distinct anterior and posterior collections of blood in relation to the lateral denticulate ligaments and the midline dorsal septum [5].

**Figure 3:**
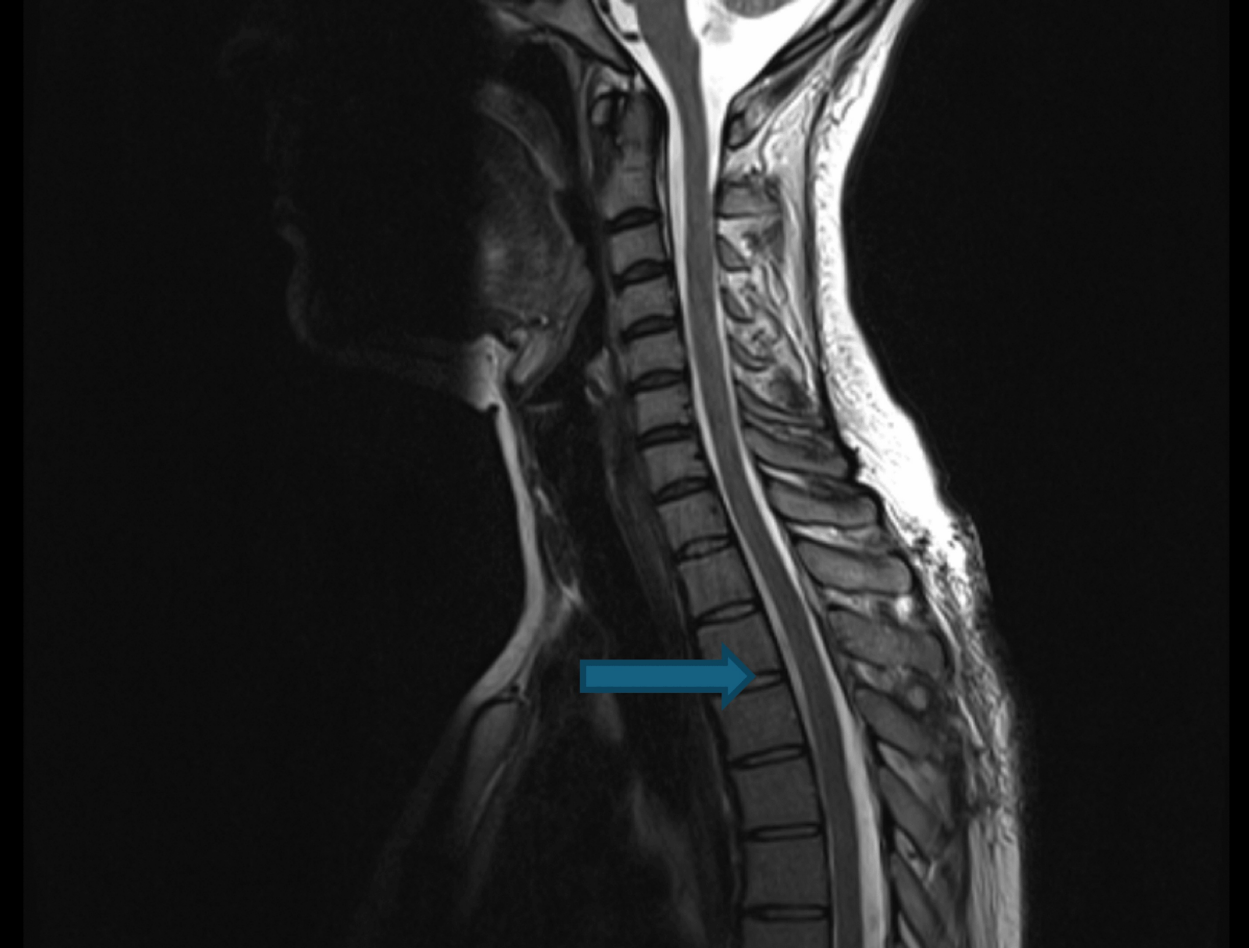
Sagittal T2-weighted MRI showing post-operative resolution of intrathecal haematoma at T3-T4. The blue arrow indicates the position of the resolved intrathecal haematoma on sagittal MRI.

**Figure 4:**
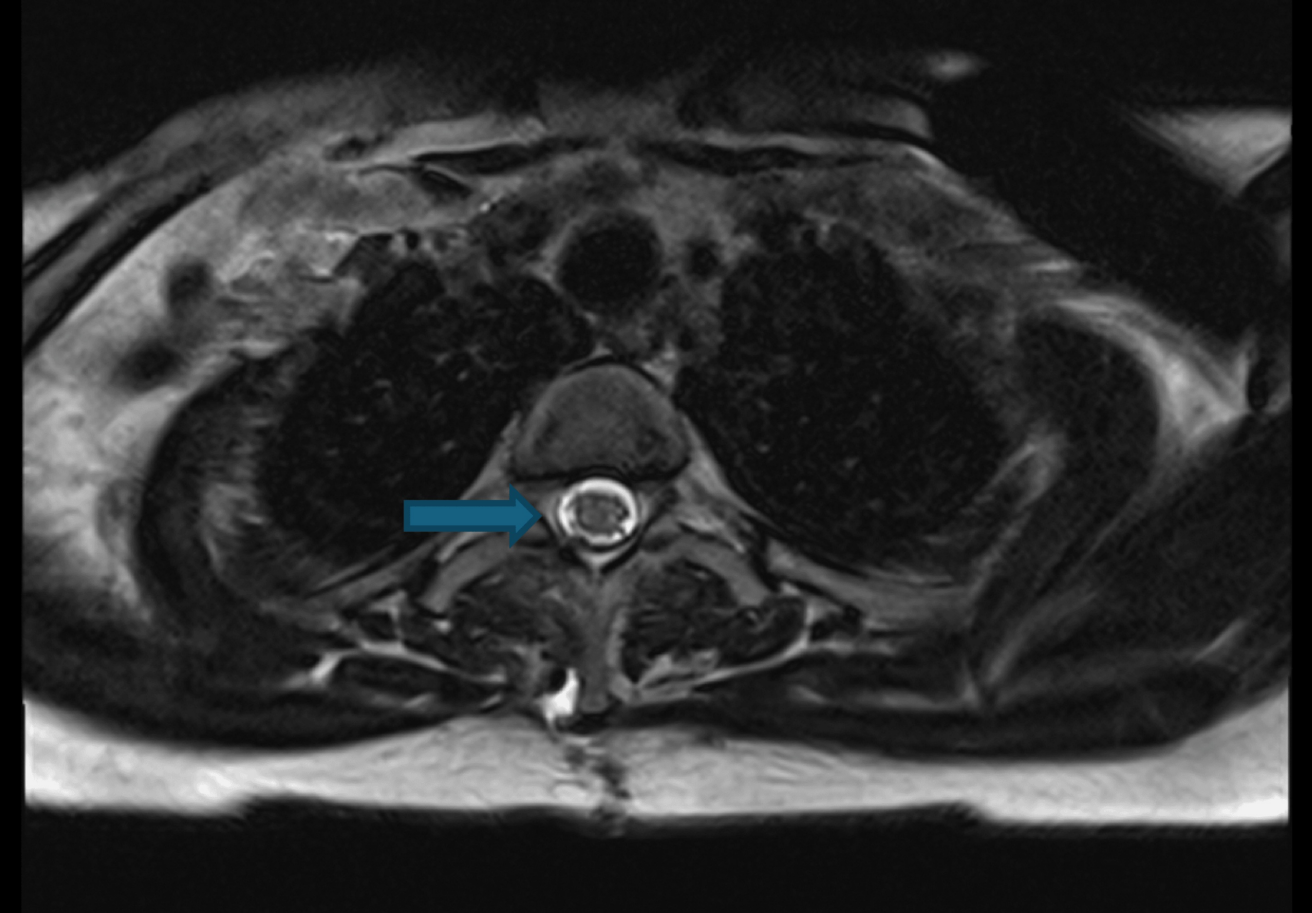
Axial T2-weighted MRI showing the post-operative resolution of intrathecal haematoma at level T3-T4. The blue arrow indicates the location of the resolved intrathecal haematoma on axial MRI.

Durotomy at T3-T4 exposed a well-organised haematoma compressing the cord, which was microsurgically evacuated. Interestingly, an addendum report was placed on the MRI images attained, stating that the haematoma was intrathecal, and there was also evidence of small volume haemorrhage in the upper cervical spine, continuous with the retroclival region, with small volume subdural haemorrhage in the posterior fossa without mass effect. Repeat dedicated MRI imaging of the brain postoperatively confirmed these findings, which required no further surgical management. Coagulation studies showed prolonged APTT and low VWF antigen (31.3), VWF:RiCoF (10), and factor VIII (47.6). The patient disclosed a forgotten childhood diagnosis of von Willebrand disease, reporting she had been lost to follow-up as she felt she had "grown out of this condition". She received 2600 units Vonvendi factor replacement prior to the procedure, and from there on out was receiving 1300 units twice a day, requiring a peripherally inserted central catheter (PICC) line due to her needle phobia. Postoperatively, neurology improved to MRC power grading 4/5 bilaterally in myotomes from L2 to S1 in the lower limbs. MRI on post-operative day 34 showed complete resolution of all haematomas, and the patient was ultimately discharged with no neurological deficit. Post-operative MRI images are depicted below in Figures 3, 4.

## Discussion

Intrathecal haematomas are an uncommon but serious neurosurgical emergency, often linked to trauma, iatrogenic causes, or coagulopathies [1,2]. Interestingly, a speculative theory in the aetiology of spontaneous intrathecal haematomas is that they are often a result of migration of bleeding from the intracranial space. This is potentially the root cause of the spinal haematoma found in this case, as there were positive findings of a retroclival subdural haematoma [6,7]. Another studied pathogenesis of this condition purports that subarachnoid haemorrhage can occur through the rupture of fragile vessels under the increase of intra-abdominal or intra-thoracic pressure on exertion, such as when completing a chest press exercise. The clotting process is impeded by this blood being diluted and redistributed by CSF, which leads to further bleeding and larger clots forming at a later stage, collecting in less dependent areas within the spinal cord [8,9]. This case uniquely involved exertion-induced intrathecal bleeding in a patient with latent von Willebrand disease, reclassified intraoperatively following real-time imaging revision. The initial MRI report was obtained from a specialist radiology registrar in training. The differentials considering the MRI images for a non-traumatic case causing paraplegia include other compressive myelopathies, such as the presence of a tumour or abscess. Non-compressive myelopathies can include transverse myelitis and multiple sclerosis (MS) as well as spinal cord infarction. Autoimmune and inflammatory causes include systemic lupus erythematosus (SLE) and sarcoids, with infectious causes, namely, human immunodeficiency virus (HIV), syphilis, and tuberculosis. Metabolic differentials can include vitamin B12 and copper deficiency [10].

With delay in diagnosis and presentation being a key factor for poor prognosis, it is important to note the factor of needle phobia, which contributed to this patient's delayed intervention. The patient's first presentation to the emergency department was on 27th December, with intervention being delayed to 30th December. From the initial assessment, the red flag of a high post-void bladder scan warranted further investigation with admission; however, the patient protested this, stating clearly it was the prospect of a blood test, which influenced her to self-discharge against medical advice. A systematic review of needle phobia depicted this to be as high as up to 30% in young adults and often resulted in avoidance of treatment [11].

From the literature, subdural spinal haematomas can present with a sharp knife stabbing pain at the site of haemorrhage, classically termed “Coup de poignard of Michon”. This term was coined by the French physician Paul Michon in 1928, which translates to the “strike of the dagger” [12]. This can be followed by a pain-free period of a few hours up to days, which is then normally followed by progressive neurological deficit below the level of the affected spinal cord. This causes a variation in the time to initial presentation from the time of bleeding, from a few hours to days [13]. On radiological assessment, MRI is the preferred modality for assessing the location, extent, morphology, and progression of a haematoma, as well as for monitoring patients over time, similar to its role in evaluating other causes of spinal cord compression [14]. Management options include conservative and surgical options. Conservative management is reserved for patients with minimal neurological deficits, especially within the context of a poor physiological reserve to withstand surgical insult, with close monitoring to observe for resolution of spinal haematoma [15]. The mainstay of management is surgical decompressive laminectomy and haematoma evacuation, with the literature reporting this to be the choice of treatment in nearly 80% of cases [3].

Von Willebrand disease, the most common inherited coagulopathy, can go undiagnosed until significant stressors unmask it [16]. The patient was found to have type 2 von Willebrand disease, an autosomal dominant variant characterised by defective von Willebrand factor, translating into a moderate to severe bleeding risk. Chronic management in stable non-bleeding patients constitutes a trial of desmopressin, which can encourage the release of von Willebrand factor from endothelial cells. However, in concerning bleeding scenarios, such as this case, administration of recombinant von Willebrand factor, such as Vonvendi, is the mainstay of treatment [17]. A systematic review of 122 cases of acute spontaneous spinal subdural haematomas, nontraumatic and non-iatrogenic in nature, found that 48% of patients had a coagulopathy. They recorded a slight propensity for female patients, with the most common location being the thoracic region of the spine. Although motor and sensory symptoms were part of the common presenting picture, 6% of patients presented initially with radiculating pain without any focal neurological deficit [3]. The role of a detailed history and a high index of suspicion is paramount. Furthermore, this case emphasises caution with red flag symptoms in back pain and the necessity of surgical exploration even when imaging appears conclusive.

## Conclusions

Intrathecal spinal haematomas must be considered in patients presenting with back pain accompanied by red flag symptoms, regardless of trauma history or known coagulopathy. A previously undocumented bleeding disorder should not be ruled out without appropriate laboratory evaluation. The patient required enhanced haematological follow-up with repeat coagulation and von Willebrand screening, with familial screening also offered. The patient was advised not to participate in any dangerous or strenuous activity. This case underscores the importance of a thorough history, interdisciplinary imaging review, and timely surgical decompression, with early involvement of haematology when considering unknown bleeding disorders. Prompt recognition and intervention are vital to optimise neurological recovery and prevent permanent disability.
